# Health impact Assessment practice in Wales: factors conditioning its success

**DOI:** 10.1093/eurpub/ckac129.101

**Published:** 2022-10-25

**Authors:** L Green

**Affiliations:** 1 HIA, Public Health Wales, Wrexham, UK; 2 Healthy Urban Environments, University of West of England, Bristol, UK

## Abstract

Historically, Public Health Institutes (PHIs) were formed to address emergency public health and environmental health related challenges for example, infectious disease outbreaks and sanitary conditions which will affect health. The COVID-19 pandemic has demonstrated the importance and value of PHIs and the specific knowledge and expertise sat within them in the 21st century in relation to critical health issues. However, PHIs have also the potential expertise to look at the wider determinants of health and how they might affect population health and inequalities. In doing so, they play a critical role to advocate for Health in All Policies (HiAP) and Health Impact Assessment (HIA) by engaging with decision makers from health and non-health sectors and providing evidence and health intelligence. Public Health Wales (PHW) has conducted very complex studies as the HIA of the impact of Brexit on the population of Wales, which was praised as very useful by the Welsh Government and local decision makers at a time when, otherwise, little robust evidence-based information was available. Other positive HIA experiences are the recently published HIA about impacts of climate change or COVID-19 pandemic. Those achievements were possible thanks to the establishment of a specific unit dedicated to HIA within PHW, and to the political support and resources. This has built awareness raising and trust in HIA as a tool to support decision making from all sectors in Wales, enabled also by much training and stakeholder participation.

